# Medico-Legal Considerations of Seat Belt Syndrome With Severe Abdominal Trauma: A Case Report and Literature Review

**DOI:** 10.7759/cureus.90902

**Published:** 2025-08-24

**Authors:** Stefania Ungureanu, Camelia-Oana Muresan, Alexandra Enache, Veronica Ciocan, Emanuela Stan, Raluca Dumache, Georgiana-Denisa Gavrilita, Alina Cristina Barb

**Affiliations:** 1 Department of Neuroscience, Discipline of Forensic Medicine, Bioethics, Deontology, and Medical Law, Victor Babeș University of Medicine and Pharmacy of Timișoara, Timișoara, ROU; 2 Department of Legal Medicine, Timișoara Institute of Legal Medicine, Timișoara, ROU; 3 Department of Neuroscience, Ethics and Human Identification Research Center, Victor Babeș University of Medicine and Pharmacy of Timișoara, Timișoara, ROU; 4 Doctoral School, Victor Babeș University of Medicine and Pharmacy of Timișoara, Timișoara, ROU; 5 Department of Microscopic Morphology/Histology, Victor Babeș University of Medicine and Pharmacy of Timișoara, Timișoara, ROU; 6 Department of Clinical Oncology, OncoHelp Hospital, Timișoara, Timișoara, ROU

**Keywords:** abdominal blunt trauma, legal medicine, road-traffic accident, seat belt sign, seat belt syndrome

## Abstract

The seat belt sign (SBS) represents a unique injury profile consisting of ecchymosis and/or abraded skin in the distribution of a seat belt, evident after a road traffic accident (RTA). When accompanied by a radiological sign that illustrates internal injury, it is referred to as seat belt syndrome (SBSy). This case report highlights the importance of recognizing SBS and SBSy in medico-legal (ML) practice and helps physicians understand injury patterns found in seat belt wearers after an RTA. Accurate assessment of this trauma enables ML physicians to understand the mechanisms of the injuries and their life-threatening potential. We present the case of a 20-year-old male student who, while seated in the front seat wearing a seat belt, sustained injuries in an RTA and subsequently sought a medico-legal certificate (MLC) at the Timisoara Institute of Legal Medicine. The ML evaluation involved a detailed examination of external injuries and a thorough analysis of medical records. The most important aspect of this case is the ML approach to SBS, which has not been previously documented. We propose a three-step algorithm to facilitate accurate ML conclusions. This report provides valuable information to ML physicians and enhances the assessment of traumatic injuries in RTA victims by establishing a framework for addressing SBS.

## Introduction

Medico-legal (ML) practice in Romania regarding road traffic accidents (RTAs) encompasses thanatology, which analyzes causes of death and traumatic injuries, and clinical legal medicine, which assesses living patients [1-5]. Victims may present to the ML practice for a medico-legal certificate (MLC) that can be obtained within the first 30 days after the traumatic event, with the examination conducted at their request [1,4,5]. If they are unable to come to the practice in the first month after the accident, the MLC cannot be issued later [1,4,5]. Therefore, if the injuries from an RTA are severe and the patient requires hospitalization, police investigators will ask ML physicians to examine the patient in the hospital [1,4,5]. In such instances, the examination is not performed at the request of the patient, but of the police. If this is the case, the ML document is called a medico-legal report (MLR) [1,4,5].

The ML examination, in the context of either an MLC or an MLR, mostly assesses the severity of road traffic injuries and their mechanisms [1]. Moreover, in cases of life-threatening injuries, the ML physician must specify in the conclusions of the ML document (MLC or MLR) that the injuries threatened the patient’s life. This is necessary for the police to correctly assess injury severity according to the Romanian Penal Code. However, it is understandable that most injuries treated by ML physicians in the context of MLCs are not severe or life-threatening, as such patients often require long-term hospitalization or bed rest and cannot come to the ML institute for assessment within a month after the car crash [1-5].

Although seat belts aim to prevent significant injuries and the overall number of deaths from RTAs has decreased because of them, the force applied to the body by the restraining effect of the belts raises the probability of intra-abdominal injuries [6-9]. The seat belt sign (SBS) consists of a clinical sign (ecchymosis and/or abraded skin in the distribution of a seat belt), evident after an RTA, while seat belt syndrome (SBSy) represents a radiological sign that illustrates internal injury in addition to SBS [9-12]. In summary, SBS refers to a clinical sign, while SBSy refers to SBS combined with a radiological sign [9-12].

The presence of SBS should heighten clinical suspicion for occult injuries to internal structures, particularly hollow viscera and mesentery [12-15]. Aortic injuries, intestinal perforations, mesenteric tears, and thoraco-lumbar vertebral burst fractures are often encountered in SBSy [16]. Chest injuries are caused by the restraining influence of the diagonal portion of the seat belt. They consist of soft tissue injuries to the chest wall, rib fractures, and sternal fractures, and may be associated with myocardial contusion [17,18]. Compression by safety devices can sometimes contribute to cardiac injuries in RTAs. The use of seat belts and airbags can result in mechanical chest compression, which may lead to cardiac contusions or other forms of blunt cardiac trauma [19]. In addition, SBS can aid in injury prediction and guide clinical practice, and may also offer corroborating data regarding occupant kinematics derived from vehicle movements, depending on the crash type [8].

In this paper, we illustrate the ML assessment of an RTA victim who came to the Timisoara Institute of Legal Medicine for an MLC 20 days after the car crash. He was a front-seat passenger wearing a seat belt. Analysis of the patient’s medical documents unexpectedly revealed that he sustained severe abdominal trauma requiring emergency surgery. This case is notable for the patient’s specific pattern of life-threatening injuries, as well as their mechanism.

This case report highlights the importance of recognizing the SBS and SBSy in ML practice and helps physicians understand injury patterns in seat belt wearers after an RTA. Moreover, the proposed three-step algorithm for a correct assessment of this trauma helps the ML physician understand the mechanisms of the injuries and their life-threatening potential.

## Case presentation

A 20-year-old male student was involved in an RTA as a front-seat passenger who was wearing a seat belt. The victim came to the Timisoara Institute of Legal Medicine to obtain an MLC 20 days after the RTA occurred. The ML approach consisted of an examination of the patient’s external injuries and a thorough analysis of the medical documents.

External examination

**Figure 1:**
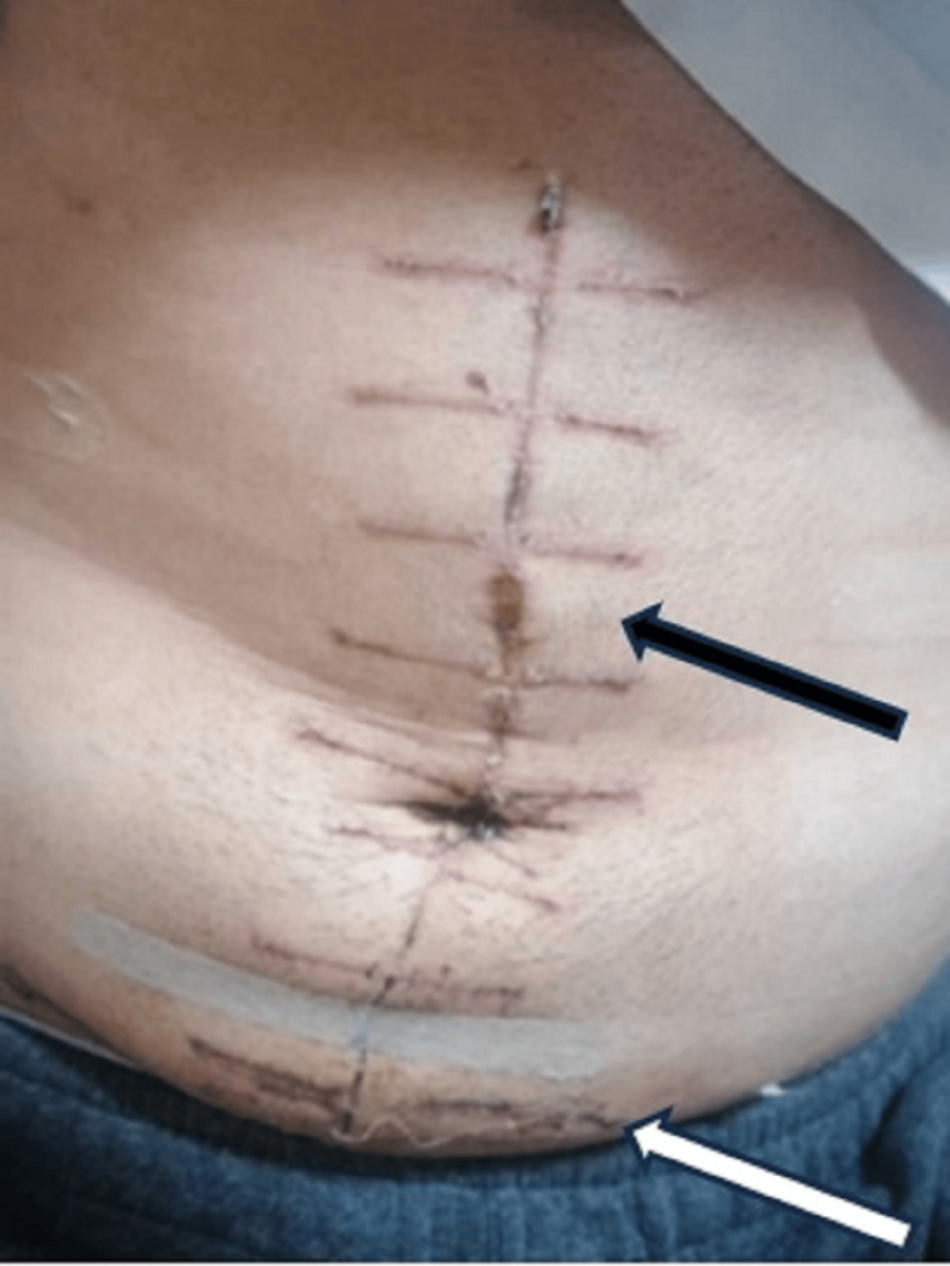
External examination of the victim’s body: anterior abdominal wall with a vertical postoperative wound (black arrow) and a classic horizontal lower-abdominal abrasion consistent with the lap belt position (white arrow).

The external examination revealed a healing vertical postoperative wound located on the anterior abdominal wall measuring 18 cm in length, and a classic horizontal lower-abdominal abrasion measuring 20 cm x 1.5 cm, consistent with the lap belt position (Figure 1). There were also small abrasions on the lower limbs, specifically on the inner aspect of the right knee and on the left anterior thigh.

Assessment of medical documentation

The only medical document available was the hospitalization sheet, with no material on the first assessment in the Emergency Department (ED). The victim was hospitalized on the surgical ward for 10 days. On admission, the patient’s history was obtained, and a clinical examination was performed. The history revealed no relevant medical information. The patient was in critical condition but was hemodynamically stable and conscious. Vital signs were not specified. A bruise on the anterior lower abdominal wall was recognized as SBS. Deep palpation of the abdomen did not reveal rigidity or guarding, but there was tenderness in the right lower quadrant. No other abnormalities were noted during the clinical examination. Blood laboratory results are illustrated in Table 1. These parameters represent standard tests performed in all patients in Romanian hospitals.

**Table 1:** Patient's blood laboratory results.

Laboratory parameter	Result	Units	Reference range
Hemoglobin	15.7	g/dL	13.5-17.5 (male)
Hematocrit	45.3	%	40-54 (male)
Platelet count	276	x10^3^/µL	150-400
White cell count (WCC)	22.5	x10^3^/µL	4.0-11.0
Creatine kinase (CK)	236	U/L	22-198
Creatine kinase myocardial band (CK-MB)	29	U/L	5-25

On the first hospital day, both native and contrast-enhanced computed tomography (CT) scans of the head, thorax, abdomen, and pelvis were performed. The results of the CT scans are presented in Table 2.

**Table 2:** Findings of the CT scans of the head, thorax, abdomen, and pelvis.

CT scan region	Findings
Cerebral CT scan	No intracranial bleeding, ischemia, or bone lesions.
Cervical spine CT-scan	Cervical vertebral bodies aligned at the posterior margin with no fractures.
Thoracic CT scan	No pneumothorax or other lesions.
Abdominal and pelvic CT scan	Small quantity of free fluid in Morrison’s pouch, perihepatic space, perisplenic area, and between bowel loops. High-density (60 HU) free fluid within the pelvis and both paracolic gutters with fat stranding surrounding the cecum, the descending colon, and the mesentery. Some small bowel loops had mildly thickened walls. Extensive subcutaneous fat stranding/hematoma of the right flank. No free gas. No solid organ, pulmonary, or skeletal injuries. No contrast extravasation.

**Figure 2:**
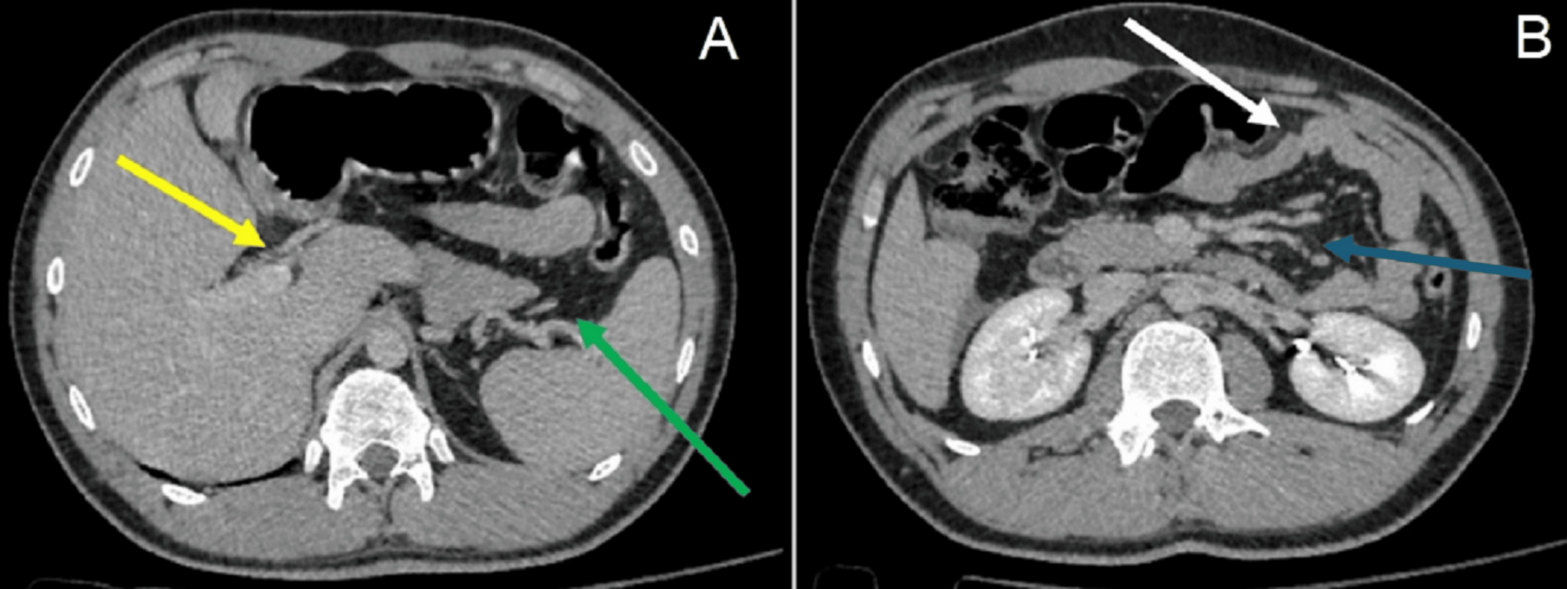
(A) CT scan of the abdomen illustrating free fluid in the peritoneal cavity in the perisplenic space (green arrow) and perihepatic space (yellow arrow). (B) CT scan of the abdomen illustrating free fluid in the peritoneal cavity between bowel loops (blue arrow) and bowel wall thickening (white arrow).

Figure 2 shows abdominal CT scan images.

**Figure 3:**
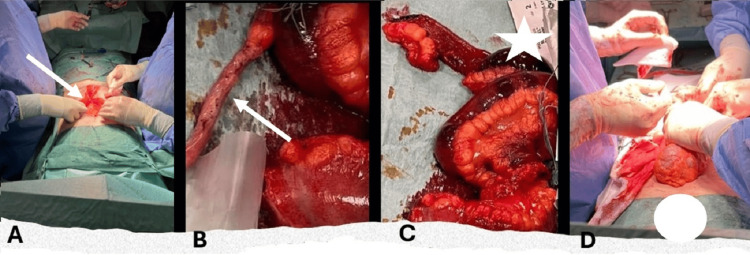
Intraoperative images: (A) laparotomy incision (white arrow); (B) the removed appendix (white arrow); (C) resection of the necrotic terminal ileum (white star); (D) healthy large bowel (white circle).

These imaging findings, the patient’s symptoms, and the presence of SBS mandated exploratory surgery. The patient underwent a laparoscopy that revealed high-volume hemoperitoneum. This was followed by abdominal laparotomy that demonstrated a 15-cm mesenteric tear of the terminal ileum, ileal necrosis, hematomas in the left paracolic gutter and the mesocolon of the sigmoid colon, and serosal tears of the sigmoid colon. The patient underwent resection of the terminal ileum with ileocecal anastomosis and suturing of the sigmoid serosal tears. Drain tubes were placed in the left paracolic gutter and in the pouch of Douglas. An appendectomy was also performed. Figure 3 illustrates the intraoperative findings. The postoperative period was uneventful, and the patient was discharged on the eighth postoperative day.

ML conclusions

Following analysis of medical documentation and external examination, the ML assessment concluded that the traumatic injuries were consistent with the reported motor vehicle accident. Moreover, the injuries represented a threat to the victim’s life. The injury pattern established that the victim was a front-seat passenger wearing a seat belt, with injuries directly attributable to seat belt compression.

Second ML examination

**Figure 4:**
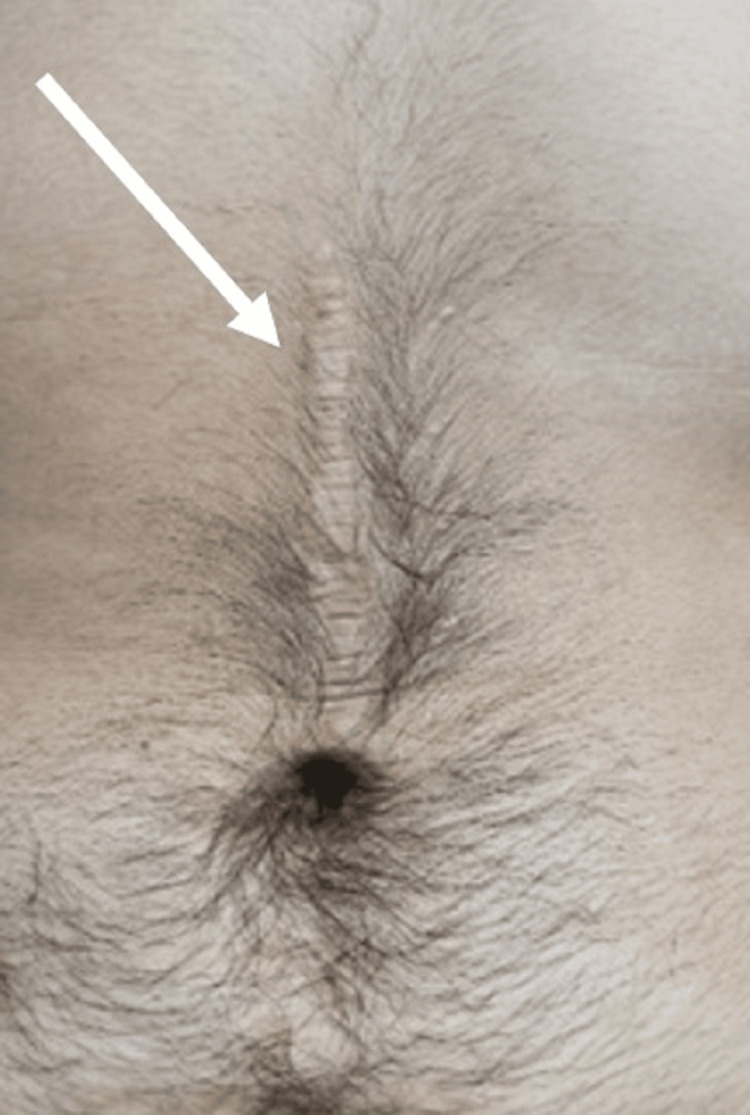
Reassessment 18 months after the accident: postoperative scar on the abdominal wall (white arrow).

Eighteen months after the accident, the police requested a second ML assessment of the victim to establish long-term complications of the initial injuries. In this case, an MLR was issued. The patient was examined, and a surgical consultation was required. New medical documents with postoperative check-outs established that the patient presented with chronic diarrhea as a sequela of his injuries. Clinical examination revealed a postoperative scar on the abdominal wall (Figure 4).

**Figure 5:**
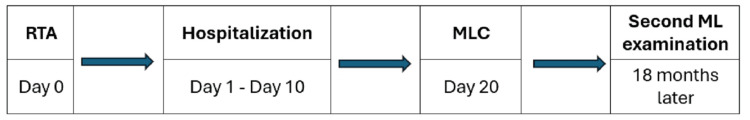
Timeline of the presented case. RTA: road traffic accident; MLC: medico-legal certificate; ML: medico-legal

For better understanding, in Figure 5, we provide a timeline of the presented case.

## Discussion

When evaluating serious injuries in RTA victims during MLCs, understanding accident kinematics and injury mechanisms is essential [2,4]. In this case, discovering that the patient wore a seat belt explained both the injury pattern and mechanism. No existing study has assessed SBS from an ML point of view, and this case report may aid physicians in recognizing this condition and in establishing the correct ML conclusions. We performed a search through the PubMed library for the keywords “seat belt sign,” and we came up with 105 results in the period 2000-2025. We could not find any articles regarding the ML or forensics approach to the SBS, only surgical and radiological interpretations highlighting the importance of the early recognition of the SBS. At our forensic institute, this injury pattern is rare in the context of MLCs, so recognizing SBS and SBSy is key to understanding injury mechanisms.

The first step in ML assessment for MLCs is obtaining the patient history: the incident details (RTA), timing (within 30 days of examination), and available medical documentation [4,5].

The second step in the assessment is to read all medical documents to understand the victim’s internal traumatic injuries [4,5]. ML assessment of medical records for MLC does not evaluate document quality, diagnostic algorithms, laboratory tests, or surgical approaches. We simply gather information to establish injury severity for our ML conclusions [5]. In this case, the patient presented with SBS and SBSy and was rapidly and correctly diagnosed, and therefore, emergency surgery was performed. These types of intestinal injuries require proper treatment, as they are linked to high death and morbidity rates [16]. The actions taken in the hospital were consistent with our proposed assessment algorithm: a history was first obtained, showing that the patient was in an RTA and was wearing a seat belt. This was followed by a clinical examination that revealed SBS. As ecchymosis on the abdominal wall is related to abdominal injury in almost 65% of cases (compared with 8% of cases without ecchymosis) [9,20,21], this patient was suspected of having internal abdominal injuries. Importantly, 64% of individuals with abdominal ecchymosis also demonstrate other injuries, such as those involving the abdominal wall, viscera, vertebrae, vasculature, and thorax [9,20,21]. A visible seat belt contusion is associated with a threefold higher incidence of intestinal perforation [12,16]. However, the patient in this study did not present with intestinal perforation but rather with a mesenteric tear, ileal necrosis, and serosal tears of the sigmoid colon. Clinical examination revealed abdominal pain or tenderness combined with positive SBS, which, according to the literature, increases the likelihood of traumatic bowel injuries [10,22]. CT investigation of the abdomen and pelvis demonstrated these injuries. The patient’s history correlated with the clinical and paraclinical examinations and established the correct diagnosis. In these types of cases, the correct diagnosis is even more difficult because delayed onset of nonspecific symptoms can occur, and they require rapid recognition by emergency services and evaluation in specialized trauma centers [21,23]. Some injuries require emergency operative treatment within hours [21].

In this case, we did not have the medical documentation from the patient’s initial assessment in the ED. These documents are usually necessary to allow ML physicians to evaluate the patient’s presenting state and assess injury severity [5]. However, although it would have been beneficial to have the whole picture, the patient’s diagnosis and presenting injuries correlated with the emergency surgery, and intraoperative findings were enough to establish that the injuries were life-threatening. In most cases, we evaluate the Glasgow Coma Scale score, blood pressure, oxygen saturation, and any clinical and paraclinical signs showing deterioration that necessitates emergency medical care [4,5]. This assessment is part of the ML evaluation of SBS. However, in the present case, we consider that these evaluations were unnecessary, as all the information we needed was on the hospitalization sheet. In other cases, the lack of ED documentation would have been considered a limitation. In cases when we lack the necessary documentation to establish the ML conclusions, we request that the hospital send us all the patient’s medical documents, and the ML assessment is delayed until we have evaluated them [5].

The gold standard investigation in trauma is the contrast-enhanced CT scan of the abdomen and pelvis, which is highly sensitive for identifying significant intra-abdominal injuries [15,16]. While a negative CT scan excludes operative hollow viscus injuries, these are often occult on CT; as such, performing repeat abdominal examinations and CT scans may be necessary [24-26]. In this case, however, the abdominal and pelvic CT scan results were decisive for the diagnosis. Intraperitoneal fluid is often the only indicator of a substantial bowel injury on CT evaluation, while suggestive signs for bowel and mesenteric injuries include fat stranding and bowel wall thickening [27,28]. Specific signs of mesenteric injuries on CT include mesenteric avulsion resulting in ischemic changes of the intestinal loop, active bleeding, and mesenteric hematoma [27]. The presence of free gas in addition to pelvic free fluid and thickened bowel loops should increase suspicion for internal injury [25]. In the present case, the CT scan showed no free gas but did reveal free fluid in the peritoneal cavity, in addition to fat stranding and thickening of the small bowel walls.

The treatment course following RTAs varies from conservative to operative, depending on the injured organ and the general condition of the patient [26]. Traumatic bowel and mesenteric injuries, such as perforation or active mesenteric bleeding, require operative management, and emergency laparoscopy is now considered an excellent alternative to abdominal laparotomy in select trauma cases [27,29]. However, hemodynamic instability with reasonable clinical suspicion of an underlying injury to the abdominal organs, positive abdominal signs on clinical examination, positive diagnostic imaging, and abdominal findings by laparoscopy are all indications for laparotomy [27]. In the presented case, the victim underwent a laparoscopy followed by a laparotomy. Significant devascularization injuries to the small bowel require emergency operative interventions, emphasizing that early diagnosis is crucial, as delays can lead to severe complications and adverse outcomes [30-32]. The terminal ileum is prone to injury since it is a relatively fixed area of the bowel [33]. The sigmoid colon is also vulnerable to tearing despite having a relatively mobile mesocolon with rich vasculature [34]. This explains the injuries sustained by our patient. An isolated colon injury following blunt abdominal trauma is rare [27]. Our case represents an example of this uncommon injury pattern. Vailas et al. state that in most cases, traumatic injuries to the mesentery are isolated [27]. This was not true in our patient, as the mesenteric injury was not the only one present.

The third step in the evaluation of victims of RTAs in an ML context is to examine the victims' bodies to identify external injuries [4,5]. Depending on the type of crash, some victims may present with only minor cuts and bruises, while others sustain severe injuries requiring surgical intervention [2-4]. Various conclusions can be drawn depending on the distribution of the abdominal wall injuries: if the patient presents with bruises or abrasions in the distribution of a seat belt, this is noted as SBS [9-12]. If SBS is present, a surgical consult may be needed [7], and the patient can be sent to the hospital for investigations [5], but only if symptoms are present. This is an option for patients who request an ML examination soon after the RTA and before going to the hospital [4,5]. In front-seat passengers, other injury patterns may be associated with SBS, such as bruises or abrasions on the knees and patella; femur fractures (due to impact with the dashboard); head and facial injuries, including bruises, abrasions, cuts, skull fractures, nasal fractures, and mandibular fractures (due to the impact with the windshield); and burns from airbag deployment [2-5].

After the assessment steps are complete, the ML conclusions can be formulated to answer the following questions: What injuries were sustained? Were the injuries potentially life-threatening? What are the mechanisms of the injuries? Can the injuries be the result of an RTA? What is the severity score of the injuries? The questions outlined above constitute the standard inquiries routinely posed by police investigators [1,3-5].

Apart from the initial ML examination, we are sometimes required to perform a second evaluation [4,5], as in the case presented here. This refers to the long-term complications (sequelae) [4,5]. Therefore, we propose another question: What are the long-term complications of the initial injuries?

Not many studies have assessed the long-term complications that can arise from seat belt injuries with abdominal trauma. One case report described adhesions resulting in chronic intermittent intestinal obstruction seven years after the initial trauma [12]. However, our literature search revealed no reports of chronic diarrhea following intestinal resection for abdominal trauma in the context of seat belt injuries. Mechanisms that cause diarrhea include impaired water absorption, microinflammation in the digestive tract, mucosal barrier dysfunction, malabsorption of bile acids, intestinal motility disorders, malabsorption of dietary components, and intestinal bacterial abnormalities [35-40]. In the present case, we believe that chronic diarrhea may have been due to resection of a small portion of the intestine, short bowel syndrome, bile acid malabsorption after ileal resection, or adhesion-related issues. However, the surgical consultations provided no definitive explanation. Regardless, the second ML examination concluded that chronic diarrhea was a complication of the injuries sustained in the RTA.

**Figure 6:**
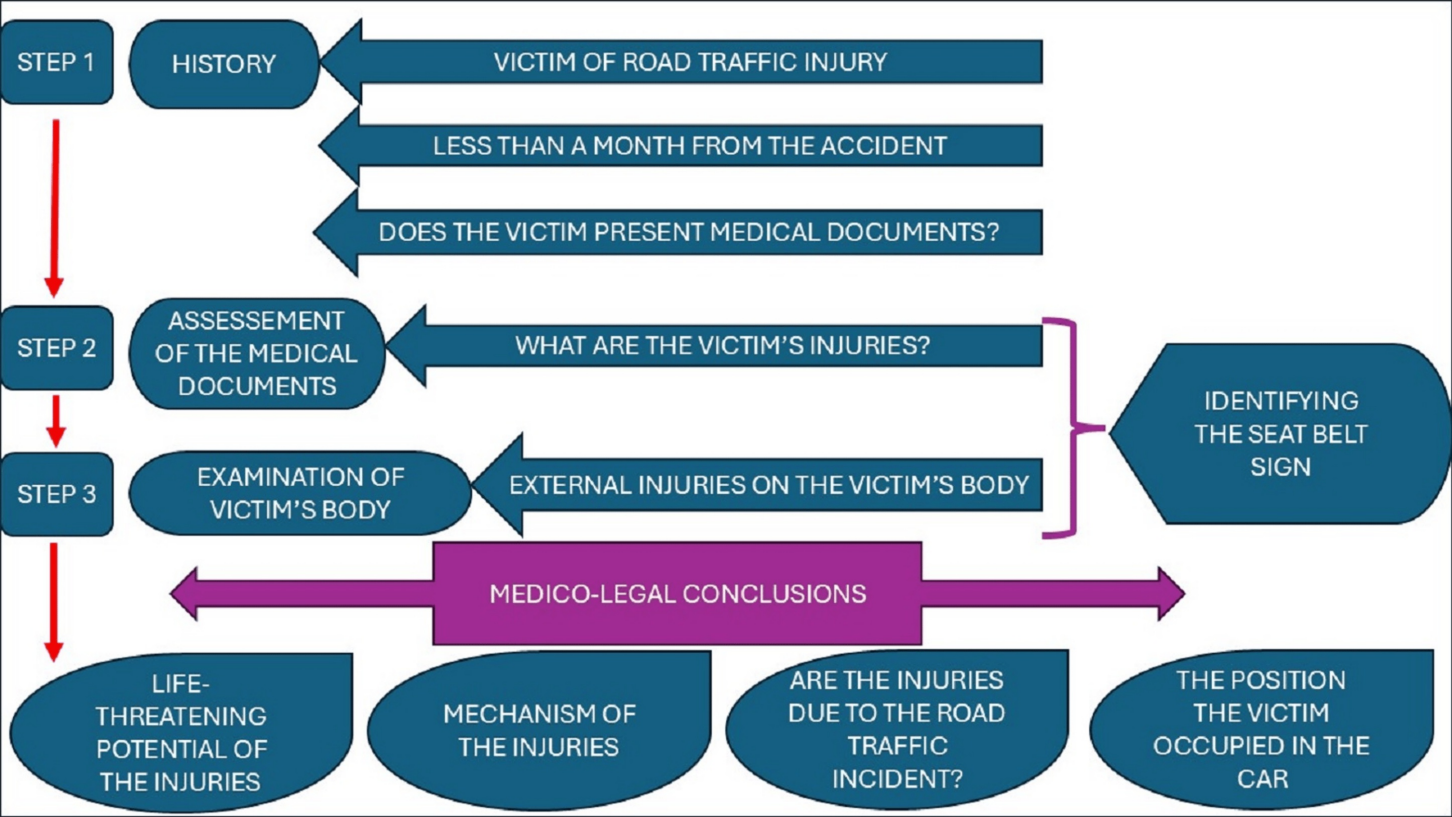
Author-proposed three-step algorithm for the ML approach to SBS for an MLC. For example, if we apply the proposed algorithm to our case, the first step reveals that the victim was involved in an RTA as a front-seat passenger who wore a seat belt, the victim came to the Timisoara Institute of Legal Medicine 20 days after the RTA happened, in time for an MLC, and provided medical documents. In step 2, with analysis of the medical documentation, we acknowledge the traumatic injuries the victim sustained in the RTA. By applying step 3, the external examination of the victim’s body reveals the SBS. Therefore, by taking all three steps, we can arrive at the medico-legal conclusions: the life-threatening potential of the injuries, how the injuries occurred in the context of an RTA, and the position the victim occupied in the car. RTA: road traffic accident; MLC: medico-legal certificate; ML: medico-legal; SBS: seat belt sign Credit: Image created by the authors.

After evaluating this patient with seat belt trauma, we developed a three-step algorithm outlining the ML approach to SBS (Figure 6). However, we acknowledge a limitation regarding this algorithm. It needs validation on a larger series of cases for a strong applicability, seeing that it is currently based on a single case, which may limit generalizability.

## Conclusions

This case report describes a victim of an RTA with abdominal trauma associated with SBS, which was promptly and correctly diagnosed and treated. The most important aspect this case presentation illustrates is the ML approach to SBS, which is not described in other medical literature. For this purpose, we proposed a three-step algorithm that leads to the correct ML conclusions. The life-threatening potential of injuries assists police investigations by clarifying the legal severity of accidents. Identifying injuries in medical documents or on the victim’s body helps establish injury mechanisms and the victim’s position in the vehicle. This case report provides valuable information to ML physicians for assessing traumatic injuries in RTA victims by describing the approach to SBS.
